# The *BCL2* -938C>A Promoter Polymorphism Is Associated with Risk for and Time to Aseptic Loosening of Total Hip Arthroplasty

**DOI:** 10.1371/journal.pone.0149528

**Published:** 2016-02-16

**Authors:** Patrick Stelmach, Christian Wedemeyer, Lena Fuest, Gina Kurscheid, Thorsten Gehrke, Stefanie Klenke, Marcus Jäger, Max D. Kauther, Hagen S. Bachmann

**Affiliations:** 1 Institute of Pharmacogenetics, University Hospital Essen, Essen, Germany; 2 Department of Orthopaedics and Trauma Surgery, University Hospital Essen, Essen, Germany; 3 Department of Joint Surgery, Helios ENDO-Klinik, Hamburg, Germany; 4 Department of Anaesthesiology and Intensive Care, University Hospital Essen, Essen, Germany; Universitat Pompeu Fabra, SPAIN

## Abstract

Aseptic loosening is a major cause of revision surgery of total hip arthroplasty (THA). Only few host factors affecting aseptic loosening have been identified until now, although they are urgently needed to identify and possibly treat those patients at higher risk for aseptic loosening. To determine whether the functional single nucleotide polymorphism (SNP) c.-938C>A (rs2279115), located in the promoter region of the *BCL2* gene has an impact on aseptic loosening of THA we genotyped and analyzed 234 patients suffering from aseptic loosening and 231 patients after primary THA. The polymorphism is associated with risk for aseptic loosening with the CC genotype at highest risk for aseptic loosening, Odds Ratio CC vs. AA 1.93, 95%CI 1.15–3.25, p = 0.013. In contrast, low risk AA genotype carriers that still developed aseptic loosening showed a significantly shorter time to aseptic loosening than patients carrying the C allele (p = 0.004). These results indicate that the *BCL2* -938C>A polymorphism influences the occurrence and course of aseptic loosening and suggests this polymorphism as an interesting candidate for prospective studies and analyses in THA registers.

## Introduction

Aseptic loosening is a major cause for revision surgery after total hip arthroplasty (THA). Studies suggest that it accounts for more than 55% of all failures in THA [1]. Only few host factors affecting aseptic loosening have been identified until now [2, 3]. In recent years genetic variations in different genes were investigated for putative impact on aseptic loosening [4]. These candidate gene approach studies analyzed different signaling cascades, e.g. inflammatory cytokine genes, but not the apoptosis pathway. Results overall indicate that genetic variants and susceptibility play a relevant role in aseptic loosening [5]. To find new and reliable host factors it is necessary to understand the pathophysiology and identify mechanisms triggering aseptic loosening. One important mechanism is the induction of a non-infectious inflammatory cascade caused by wear debris of the implanted artificial joint [6]. Studies on tissue and the periprosthetic interface membrane surrounding aseptically loosened hips show that macrophages play a key role in this cascade. They take wear particles up and release a number of cytokines and pro-inflammatory factors that activate osteoclasts [7]. Furthermore, different pro-apoptotic markers are highly expressed in macrophages of the aseptic interface membrane [8]. Consistently, macrophage apoptosis occurs frequently in interface membranes and is associated with periprosthetic osteolysis [9]. Therefore, apoptosis is widely discussed as a specific target for therapeutic modulation of aseptic loosening [10].

Among other factors, apoptosis is regulated by pro- and anti-apoptotic proteins of the Bcl-2 family [11]. Bcl-2 itself is an anti-apoptotic protein, which can be down regulated e.g. by DNA-damage-inducible transcript 3 (DDIT3). Yang et al. showed an increased expression of DDIT3 in macrophages of the interface membrane [10]. Consistently, only basal expression levels of Bcl-2 are detectable in macrophages of the interface membrane [12].

The promoter region of the *BCL2* gene harbors a functional single nucleotide polymorphism c.-938C>A (rs2279115) [13]. Alleles of this polymorphism lead to altered transcription factor binding and altered basal promoter activity in different cell lines [14, 15]. The AA genotype is associated with increased Bcl-2 expression on the mRNA as well as protein level in white blood cells of healthy subjects and in different tumor tissues [15–18]. Furthermore, -938C>A genotypes have an impact on risk [19, 20], outcome [21, 22], and drug response in different diseases [18, 23].

Recently, we investigated putative effects of polymorphisms, among them the *BCL2* -938C>A polymorphism, in a preliminary study comprising 87 patients suffering from aseptic loosening [24]. We were not able to show any significant difference with regard to time to aseptic loosening. However, -938C>A genotype distribution of aseptic loosening patients differed significantly from healthy controls, the CC genotype being associated with highest risk for aseptic loosening. The question remains whether this deviation biased the preliminary analysis or might be an indicator of a risk modulation by -938C>A genotypes. To answer this question we performed an independent second study with an increased number of patients and a more appropriate control group. This study comprises 234 patients suffering from aseptic loosening and 231 patients with primary implantation of a THA. We genotyped all patients and analyzed data with regard to risk for aseptic loosening as well as time to aseptic loosening of THA.

## Materials and Methods

### Patients

Our cohort consisted of 465 Western European patients of German ancestry. Of these, 231 had a primary implantation of a THA and 234 were operated for aseptic loosening of prosthetic hip joints at the Helios ENDO-Klinik Hamburg, Germany. Control and aseptic loosening patients were consecutively enrolled at the time of primary THA and revision surgery, respectively. For both patient groups we defined strict inclusion and exclusion criteria. For control patients we demand a primary THA due to primary osteoarthritis. For patients suffering from aseptic loosening we used the same inclusion and exclusion criteria as in our previous preliminary study [24]. Shortly, inclusion criteria were clinical, radiological and intra-surgical diagnosis of aseptic loosening after total hip arthroplasty due to primary osteoarthritis and exclusion criteria were traumatic loosening, any deep infection or the suspicion of implant infection, inflammatory disease or treatment with immunosuppressant agents in the patients’ history after THA. Exclusion of infection was carried out by microbiological swab analysis. This study was performed according to the Declaration of Helsinki and approved by the Ethics Committee of the University Hospital Essen. Written informed consent was obtained from all patients on enrollment.

### Determination of *BCL*2 Genotype

DNA was extracted from whole blood or buccal swab by using the QIAamp DNA blood mini kit (Qiagen, Hilden, Germany) following the manufacturer’s protocols. Genotypes of the *BCL2* -938C>A polymorphism were determined by Slowdown PCR and subsequent pyrosequencing as previously described [24, 25]. The following primers were used: Forward primer, 5’-GAGCAGCTTTTCGGAAAATG-3’, and biotinylated reverse primer, 5’-TATCCACGGGACCGCTTCAC-3’. The 134-bp PCR products were analyzed by pyrosequencing using the sequencing primer 5’-TCCCCGGCTCCTTCATCGTC-3’ on the PSQ96 system according to the manufacturer’s instructions (Biotage, Uppsala, Sweden). Results were analyzed using PSQ96 SNP software.

### Statistical Analysis

P values were calculated using ANOVA for trend and unpaired t-test for continuous variables and categorical variables were analyzed by χ^2^ test and χ^2^ test for trend, respectively. Odds ratios (OR) and 95% confidence intervals (CI) were calculated for comparing the risk for aseptic loosening dependent on *BCL2* genotypes. The Kruskal-Wallis test was used for comparison of median time to aseptic loosening of the different genotypes. Kaplan-Meier plots and the log-rank test were used to retrospectively evaluate the time to loosening, dependent on *BCL2* -938C>A genotype. The impact of age, BMI, and *BCL2* -938C>A genotype as prognostic factors for time to aseptic loosening were analyzed by univariate and multivariate Cox regression models. Hazard ratios (HR) and 95% CI were calculated from these Cox regression models. Control for deviation from Hardy-Weinberg equilibrium was conducted with a publically available Hardy-Weinberg equilibrium calculator [26]. Differences were regarded significant at p < 0.05. Statistical analysis was performed using SPSS 20.0 (SPSS, Chicago, IL, USA) and GraphPad Prism 6.0 (GraphPad Software, San Diego, CA, USA).

## Results

### Clinical characteristics and *BCL2* -938C>A genotypes

Clinical characteristics and -938C>A genotype distributions of patients with primary THA and of patients suffering from aseptic loosening are shown in Table 1. Genotyping of the 231 patients with primary THA revealed a *BCL2* -938C>A genotype distribution with 81 patients carrying the AA genotype, 108 heterozygous patients and 42 CC genotype carriers. Determination of -938C>A genotypes in 234 patients with aseptic loosening disclosed a genotype distribution with 58 AA genotype carriers, 118 heterozygous patients and 58 patients harboring the CC genotype. Both genotype distributions were not significantly different from Hardy Weinberg equilibrium (p = 0.569 and p = 0.896 respectively). All variables of the clinical characteristics were analyzed dependent on -938C>A genotypes in both groups. None of the variables was significantly associated with *BCL2* -938C>A genotypes. To control for differences in clinical characteristics between the groups, we compared all variables. There was no statistically significant difference in gender distribution (primary THA: 65.4% female vs. aseptic loosening: 70.5% female, p = 0.235) and age at enrollment (primary THA, age at implantation: 64.54 ± 10.9 y vs. aseptic loosening, age at replantation: 65.34 ± 12.4 y, p = 0.461) Comparison of the prognostically relevant computed value BMI revealed no statistically significant difference between the groups (primary THA: 27.31 ± 4.5 kg/m² vs. aseptic loosening: 26.94 ± 5.9 kg/m², p = 0.448).

**Table 1:** Clinical characteristics and genotype distribution in patients with primary THA and patients suffering from aseptic loosening.

		*BCL2* -938C>A genotype			
	All	CC	CA	AA	p-value
*Primary THA*					
n (%)	231	42 (18.2)	108 (46.8)	81 (35.1)	
Age at implantation (y)	64.54 ± 10.9	63.88 ± 10.4	64.45 ± 11.0	65.00 ± 11.1	0.588
Gender					
Female (%)	151 (65.4)	30 (19.9)	73 (48.3)	48 (31.8)	0.144
Male (%)	80 (34.6)	12 (15.0)	35 (43.8)	33 (41.3)	
BMI (kg/m²)	27.31 ± 4.5	26.21 ± 3.6	27.88 ± 4.7	27.16 ± 4.7	0.489
*Aseptic loosening*					
n (%)	234	58 (24.8)	118 (50.4)	58 (24.8)	
Age at implantation (y)	52.80 ± 12.8	52.22 ± 13.5	51.81 ± 11.6	55.35 ± 14.1	0.188
Age at replantation (y)	65.34 ± 12.4	65.90 ± 13.8	65.05 ± 11.4	65.39 ± 13.2	0.826
Gender					
Female (%)	165 (70.5)	41 (24.8)	88 (53.3)	36 (21.8)	0.309
Male (%)	69 (29.5)	17 (24.6)	30 (43.5)	22 (31.9)	
BMI (kg/m²)	26.94 ± 5.9	27.54 ± 9.1	26.58 ± 4.3	27.10 ± 4.3	0.683
First cup with cement (n = 212)					
no	73 (34.4)	20 (27.4)	32 (43.8)	21 (28.8)	0.780
yes	139 (65.6)	32 (23.0)	77 (55.4)	30 (21.6)	
First stem with cement (n = 213)					
no	75 (35.2)	21 (28.0)	36 (48.0)	18 (24.0)	0.538
yes	138 (64.8)	31 (22.5)	73 (52.9)	34 (24.6)	

n, number of patients. Data are numbers with percentages given in brackets and numbers with standard deviation, respectively. Categorical variables were analysed by χ^2^ for trend statistics. P values were calculated using ANOVA for trend for continuous variables.

### Risk for aseptic loosening and *BCL2* -938C>A genotypes

To determine a putative impact of the *BCL2* -938C>A polymorphism on risk for aseptic loosening, comparison of genotype distributions of both groups was performed. The A-allele frequency in the aseptic loosening group was lower than in the primary THA group (0.50 vs. 0.58). Concomitantly, the percentage of CC and CA genotype carriers was higher in the aseptic loosening group and the genotype distributions were significantly different (p = 0.011, Table 2). Pairwise comparison of patients carrying the AA and CC genotype revealed that patients with CC genotype have a significantly higher risk to belong to the aseptic loosening group (OR = 1.93, 95% CI 1.15–3.25; p = 0.013). Comparing AA with CA genotype carriers, patients with CA genotype have a higher risk to belong to the aseptic loosening group as well, but just missed the significance level (OR = 1.53, 95% CI 0.99–2.34; p = 0.051). For better comparability we re-analysed data of our previous study of aseptic loosening patients in comparison to healthy volunteers in exactly the same way as our current data (Table 2) [24]. Accordingly, current results are completely in line with our previous findings.

**Table 2:** *BCL2* -938C>A Genotype Distribution.

*This Study (Hamburg*, *Germany)*				
*BCL2*–938	Aseptic loosening	Primary THA	OR (95% CI)	p-value
	n = 234	n = 231		
AA	58	81	1	
CA	118	108	1.53 (0.99–2.34)	0.051
CC	58	42	1.93 (1.15–3.25)	0.013
	p = 0.011			
Freq. (A)	0.50	0.58		
*Previous Study (Essen, Germany; Ref. [24])*				
*BCL2*–938	Aseptic loosening	Hist. Control	OR (95% CI)	p-value
	n = 87	n = 120		
AA	16	36	1	
CA	49	63	1.75 (0.87–3.52)	0.114
CC	22	21	2.36 (1.02–5.46)	0.043
	p = 0.042			
Freq. (A)	0.47	0.56		

n, number of patients. Data are numbers. Odds Ratios (OR) and 95% Confidence intervals (CI) were calculated. P values were calculated using χ² test and χ² test for trend, respectively.

### Time to aseptic loosening and *BCL2* -938C>A genotypes

In the aseptic loosening group, time and median time to aseptic loosening were evaluated for dependency on genotypes to further analyze the impact of the *BCL2* -938C>A polymorphism on aseptic loosening. Median time to aseptic loosening showed a statistically significant association with *BCL2* -938C>A genotypes, whereas AA homozygous patients had the shortest median time to loosening (p = 0.046, Table 3). Median time to aseptic loosening was 156 months (range 3–396) for *BCL2*–938 CC homozygous patients, 150 months (range 1–431) for patients heterozygous for *BCL2*–938 and 117 months (range 1–397) for AA genotype carriers. Due to the almost similar results of CC homozygous and CA heterozygous patients we performed a combined analysis of these genotypes versus AA homozygous patients. Median time to loosening was 154 months (range 1–431) for combined CC and CA genotype carriers and significantly different from median time to aseptic loosening of AA homozygous patients (p = 0.013).

**Table 3:** Median time to aseptic loosening according to *BCL2* genotype.

		*BCL2* -938C>A genotype			
	All	CC	CA	AA	p-value
	n = 234	n = 58	n = 118	n = 58	
MTAL, months	142 (1–431)	156 (3–396)	150 (1–431)	117 (1–397)	0.046
MTAL, months	142 (1–431)	[CC+CA] 154 (1–431)		117 (1–397)	0.013

MTAL, median time to aseptic loosening; n, number of patients. Data are medians with ranges given in brackets, respectively. P values were calculated using Kruskal-Wallis test for comparison of nonparametric variables.

**Fig 1:**
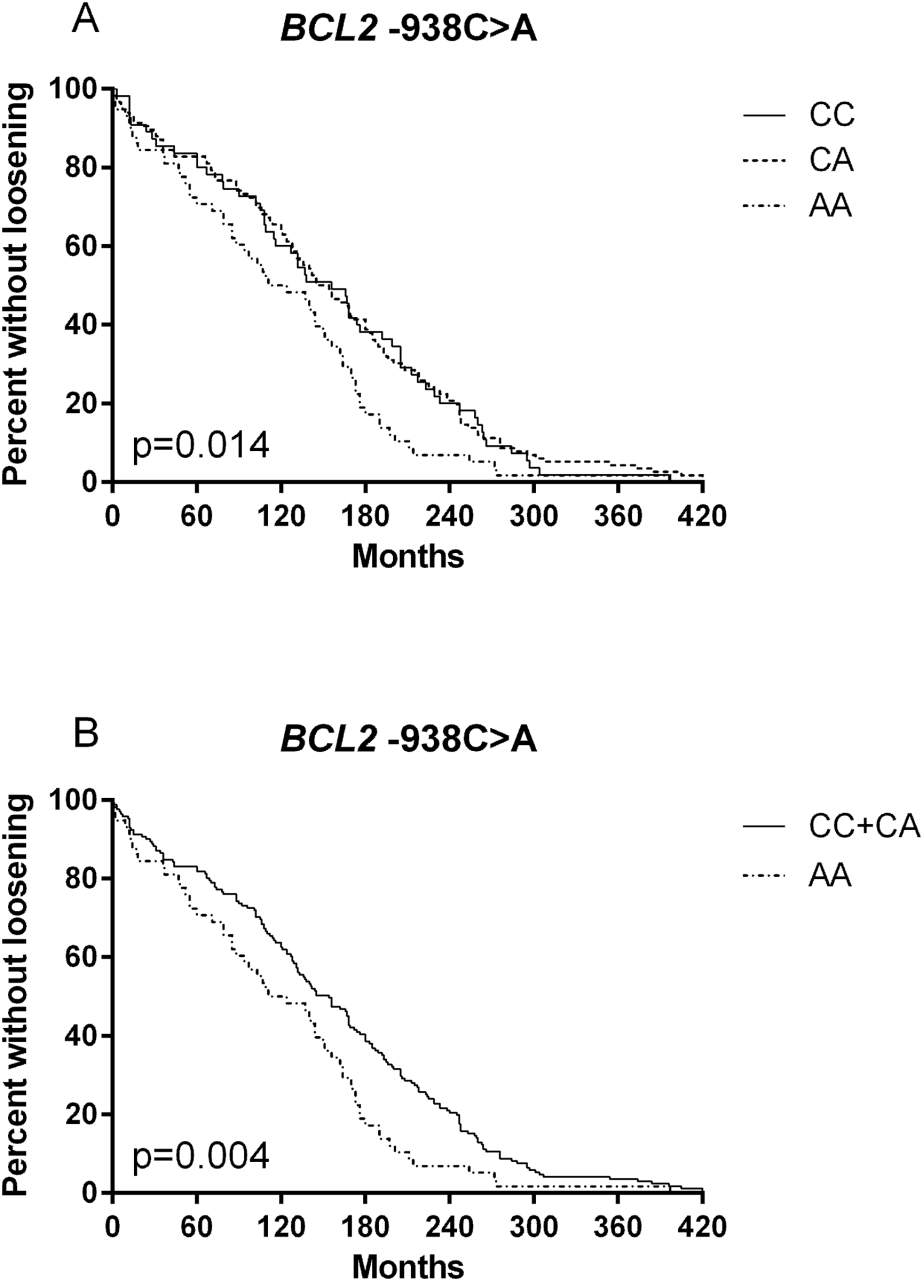
Time to aseptic loosening depending on *BCL2*–938 genotype. Time to aseptic loosening in the aseptic loosening group based on Kaplan-Meier-curves. (A) patients (n = 234), based on *BCL2* -938C>A genotype. (B) patients (n = 234) based on *BCL2* -938C>A CC+CA and AA genotype.

Kaplan-Meier curve showed a significant association of *BCL2* -938C>A genotypes with time to aseptic loosening (p = 0.014, Fig 1A). Following HRs and 95% CIs were calculated for *BCL2*–938 genotypes in univariate Cox regression analysis: CA vs. AA HR 0.631, 95% CI 0.458–0.870, p = 0.005 and CC vs. AA HR 0.665, 95% CI 0.458–0.956, p = 0.032. As potential influencing factors on time to aseptic loosening we tested age and BMI in a multivariate Cox regression analysis. Despite correcting for these variables the *BCL2* -938C>A polymorphism remained a significant factor of time to aseptic loosening (CA vs. AA HR 0.683, 95% CI 0.491–0.950, p = 0.023 and CC vs. AA HR 0.637, 95% CI 0.435–0.934, p = 0.021, Table 4).

**Table 4:** Factors influencing the time to aseptic loosening by univariate and multivariate Cox-regression analysis.

	Univariate			Multivariate		
	HR	95% CI	P	HR	95% CI	P
BCL2 -938C>A						
AA	1^a^			1^a^		
CA	0.631	0.458–0.870	0.005	0.683	0.491–0.950	0.023
CC	0.665	0.458–0.965	0.032	0.637	0.435–0.934	0.021
Age (per month)	1.003	1.002–1.004	<0.001	1.003	1.002–1.004	<0.001
BMI (per point)	1.013	0.996–1.029	0.136	1.021	1.002–1.040	0.026
BCL2 -938C>A						
AA	1^a^			1^a^		
CA+CC	0.642	0.474–0.869	0.004	0.667	0.488–0.911	0.011
Age (per month)	1.003	1.002–1.004	<0.001	1.003	1.002–1.004	<0.001
BMI (per point)	1.013	0.996–1.029	0.136	1.020	1.002–1.039	0.028

Analysis of 234 patients suffering from aseptic loosening. P values were calculated using the Cox-proportional hazard model.

^a^Reference group

In line with results of the median time to aseptic loosening analysis, individual comparison of *BCL2* -938C>A genotypes showed similar curve progression of the CC and CA genotypes in Kaplan-Meier analysis. Therefore, we computed a combined comparison of CC and CA genotypes with the AA genotype as well. As expected, combined comparison of CC and CA genotypes with AA genotype showed a significantly longer time to aseptic loosening for combined CC and CA genotypes (p = 0.004, Fig 1B). Univariate and multivariate Cox regression analysis revealed also this significant connection. Following HRs were calculated for *BCL2–*938 genotypes in univariate Cox regression analysis: CA+CC vs. AA HR 0.642, 95% CI 0.474–0.869 and multivariate Cox regression analysis: CA+CC vs. AA HR 0.667 95% CI 0.488–0.911 (Table 4).

## Discussion

Aseptic loosening of THA is one of the main causes of revision surgery after THA and a clinically relevant problem, because patients undergoing revision surgery after THA have significantly higher re-failure and complication rates and significantly lower clinical improvements compared with patients with primary THA [27, 28]. Identification of new risk and prognostic factors would be a first step to identify high risk patients and might secondly lead to improved clinical options, e.g. specific treatments for these patients. Our goal was to determine whether the *BCL2* -938C>A polymorphism influences the course and occurrence of aseptic loosening of THA. Our results indicate that the *BCL2* -938C>A polymorphism has a relevant impact on aseptic loosening of THA because genotypes were significantly associated with risk of aseptic loosening as well as time to aseptic loosening.

We observed that *BCL2* -938C>A genotype distributions of aseptic loosening patients and primary THA patients were significantly different. CC and CA genotype carriers had an increased risk for aseptic loosening of THA. Because of the limited number of patients a number of potential confounders must be considered. First, both distributions were in Hardy-Weinberg equilibrium, adequate positive and negative controls were used and re-genotyping revealed completely concordant results, which argues against genotyping errors. Second, we compared clinical characteristics of both groups and detected no significant differences with regard to age, sex and BMI. Third, the risk association and especially the calculated ORs are completely in line with previous findings in our independent preliminary study that reported similar ORs and the same gene dose effect with heterozygous patients showing an intermediate risk for aseptic loosening [24]. Furthermore, the detected association of the C allele with higher risk for aseptic loosening is in line with functional data of the polymorphism, because the C allele is associated with decreased Bcl-2 expression [15–18]. Decreased Bcl-2 expression leads to more apoptosis of macrophages which is associated with periprostetic osteolysis [9]. More periprostetic osteolysis would explain the predisposition for aseptic loosening in patients homozygous and heterozygous for the C allele of the *BCL2* -938C>A polymorphism.

To our surprise we discovered that AA homozygous aseptic loosening patients developed loosening of THA significantly earlier than homozygous and heterozygous C allele carriers. It may appear counterintuitive that the same genotype mediates the lowest risk for aseptic loosening and is associated with earlier loosening. A straightforward hypothesis would imply that increased expression of a molecular target may favor risk for as well as time to aseptic loosening. Particularly with regard to our previous preliminary study that showed no significantly different time to loosening, we cannot rule out that this is a coincidental finding. On the other hand we have no hints for a coincidental association because values of the clinical characteristic are in line with those of other studies [29, 30], variables of the clinical characteristic were not significantly associated with genotypes and the association with time to loosening remained significant in multivariate comparison. Therefore, the non-significant results of our previous preliminary study might be due to the lower sample size. Furthermore, on the molecular level the interaction of pro- and anti-apoptotic proteins is not a linear process. Three different signaling cascades are involved in apoptosis of macrophages of the interface membrane and the delicate balance between the different factors is not completely understood [10]. In addition, the polymorphism is not only present in macrophages but also in every other DNA-carrying cell. Other immune cells as well as osteoclasts and osteoblasts carry the variant and it is completely unknown whether and how all these players interact with regard to the *BCL2* -938C>A genotype on the cellular level. Therefore, one can speculate that both findings might be real, but further molecular and clinical studies are needed to clarify this. Furthermore, it should be mentioned that also other known or unknown risk factors such as the type of implants that have been used or the surgical experience may act as potentially influencing factors on time to aseptic loosening [31]. In future studies, these factors should be considered in order to exclude additional potential confounders.

In summary, our work revealed the *BCL2* -938C>A polymorphism as a putative molecular marker for aseptic loosening in THA. The AA genotype was associated with decreased risk of aseptic loosening, but in those patients that developed aseptic loosening it was associated with earlier loosening. Our results imply the polymorphism as a good candidate for further evaluation. However, it should be noted that these findings are based on a small study population of 465 patients. Therefore the observed effects in risk for and time to aseptic loosening must be considered as preliminary. Only substantially larger study cohorts could clarify whether there is a true association. Fortunately, over the past years registers for THA have been installed in many different countries. Unfortunately, most of these registers collect only clinical data [32–36]. But a few, like the Norwegian Arthroplasty Register established a DNA archive as well [37]. Therefore we would like to suggest this polymorphism as a good candidate for a first polymorphism-driven study in one of these registers.

## References

[1] Sadoghi P, Liebensteiner M, Agreiter M, Leithner A, Bohler N, Labek G. Revision surgery after total joint arthroplasty: a complication-based analysis using worldwide arthroplasty registers. The Journal of arthroplasty. 2013;28(8):1329–32. 10.1016/j.arth.2013.01.012 23602418

[2] Cherian JJ, Jauregui JJ, Banerjee S, Pierce T, Mont MA. What Host Factors Affect Aseptic Loosening After THA and TKA? Clin Orthop Relat Res. 2015. 10.1007/s11999-015-4220-2 PMC4488212 25716213

[3] Teng S, Yi C, Krettek C, Jagodzinski M. Smoking and risk of prosthesis-related complications after total hip arthroplasty: a meta-analysis of cohort studies. PloS one. 2015;10(4):e0125294 10.1371/journal.pone.0125294 25909602 PMC4409354

[4] Del Buono A, Denaro V, Maffulli N. Genetic susceptibility to aseptic loosening following total hip arthroplasty: a systematic review. British medical bulletin. 2012;101:39–55. 10.1093/bmb/ldr011 21652593

[5] Greenfield EM. Do genetic susceptibility, Toll-like receptors, and pathogen-associated molecular patterns modulate the effects of wear? Clin Orthop Relat Res. 2014;472(12):3709–17. 10.1007/s11999-014-3786-4 25034980 PMC4397765

[6] Gallo J, Vaculova J, Goodman SB, Konttinen YT, Thyssen JP. Contributions of human tissue analysis to understanding the mechanisms of loosening and osteolysis in total hip replacement. Acta biomaterialia. 2014;10(6):2354–66. 10.1016/j.actbio.2014.02.003 24525037 PMC4389682

[7] Nich C, Takakubo Y, Pajarinen J, Ainola M, Salem A, Sillat T, et al Macrophages-Key cells in the response to wear debris from joint replacements. Journal of biomedical materials research Part A. 2013;101(10):3033–45. 10.1002/jbm.a.34599 23568608 PMC3775910

[8] Landgraeber S, von Knoch M, Loer F, Wegner A, Tsokos M, Hussmann B, et al Extrinsic and intrinsic pathways of apoptosis in aseptic loosening after total hip replacement. Biomaterials. 2008;29(24–25):3444–50. 10.1016/j.biomaterials.2008.04.044 18490052

[9] Huk OL, Zukor DJ, Ralston W, Lisbona A, Petit A. Apoptosis in interface membranes of aseptically loose total hip arthroplasty. Journal of materials science Materials in medicine. 2001;12(7):653–8. 15348259 10.1023/a:1011254029864

[10] Yang F, Wu W, Cao L, Huang Y, Zhu Z, Tang T, et al Pathways of macrophage apoptosis within the interface membrane in aseptic loosening of prostheses. Biomaterials. 2011;32(35):9159–67. 10.1016/j.biomaterials.2011.08.039 21872327

[11] Volkmann N, Marassi FM, Newmeyer DD, Hanein D. The rheostat in the membrane: BCL-2 family proteins and apoptosis. Cell death and differentiation. 2014;21(2):206–15. 10.1038/cdd.2013.153 24162659 PMC3890954

[12] Landgraeber S, Toetsch M, Wedemeyer C, Saxler G, Tsokos M, von Knoch F, et al Over-expression of p53/BAK in aseptic loosening after total hip replacement. Biomaterials. 2006;27(15):3010–20. 16445975 10.1016/j.biomaterials.2006.01.006

[13] Park BL, Kim LH, Cheong HS, Cho HY, Kim EM, Shin HD, et al Identification of variants in cyclin D1 (CCND1) and B-Cell CLL/lymphoma 2 (BCL2). Journal of human genetics. 2004;49(8):449–54. 15278764 10.1007/s10038-004-0173-0

[14] El Hindy N, Bachmann HS, Lambertz N, Adamzik M, Nuckel H, Worm K, et al Association of the CC genotype of the regulatory BCL2 promoter polymorphism (-938C>A) with better 2-year survival in patients with glioblastoma multiforme. Journal of neurosurgery. 2011;114(6):1631–9. 10.3171/2010.12.JNS10478 21250804

[15] Bachmann HS, Heukamp LC, Schmitz KJ, Hilburn CF, Kahl P, Buettner R, et al Regulatory BCL2 promoter polymorphism (-938C>A) is associated with adverse outcome in patients with prostate carcinoma. International journal of cancer Journal international du cancer. 2011;129(10):2390–9. 10.1002/ijc.25904 21207420

[16] Bachmann HS, Otterbach F, Callies R, Nuckel H, Bau M, Schmid KW, et al The AA genotype of the regulatory BCL2 promoter polymorphism (938C>A) is associated with a favorable outcome in lymph node negative invasive breast cancer patients. Clinical cancer research: an official journal of the American Association for Cancer Research. 2007;13(19):5790–7. 17908970 10.1158/1078-0432.CCR-06-2673

[17] Lehnerdt GF, Franz P, Bankfalvi A, Grehl S, Kelava A, Nuckel H, et al The regulatory BCL2 promoter polymorphism (-938C>A) is associated with relapse and survival of patients with oropharyngeal squamous cell carcinoma. Annals of oncology: official journal of the European Society for Medical Oncology / ESMO. 2009;20(6):1094–9. 10.1093/annonc/mdn763 19196738

[18] Zhang C, Wu Z, Hong W, Wang Z, Peng D, Chen J, et al Influence of BCL2 gene in major depression susceptibility and antidepressant treatment outcome. J Affect Disord. 2014;155:288–94. 10.1016/j.jad.2013.11.010 24321200

[19] Dorjgochoo T, Xiang YB, Long J, Shi J, Deming S, Xu WH, et al Association of genetic markers in the BCL-2 family of apoptosis-related genes with endometrial cancer risk in a Chinese population. PloS one. 2013;8(4):e60915 10.1371/journal.pone.0060915 23637776 PMC3634058

[20] Zhang N, Li X, Tao K, Jiang L, Ma T, Yan S, et al BCL-2 (-938C > A) polymorphism is associated with breast cancer susceptibility. BMC medical genetics. 2011;12:48 10.1186/1471-2350-12-48 21457555 PMC3078853

[21] Javid J, Mir R, Mirza M, Imtiyaz A, Prasant Y, Mariyam Z, et al CC genotype of anti-apoptotic gene BCL-2 (-938 C/A) is an independent prognostic marker of unfavorable clinical outcome in patients with non-small-cell lung cancer. Clinical & translational oncology: official publication of the Federation of Spanish Oncology Societies and of the National Cancer Institute of Mexico. 2015;17(4):289–95. 10.1007/s12094-014-1226-2 25257838

[22] Park YH, Sohn SK, Kim JG, Lee MH, Song HS, Kim MK, et al Interaction between BCL2 and interleukin-10 gene polymorphisms alter outcomes of diffuse large B-cell lymphoma following rituximab plus CHOP chemotherapy. Clinical cancer research: an official journal of the American Association for Cancer Research. 2009;15(6):2107–15. 19276283 10.1158/1078-0432.CCR-08-1588

[23] Kunkele A, Grosse-Lordemann A, Schramm A, Eggert A, Schulte JH, Bachmann HS. The BCL2-938 C > A promoter polymorphism is associated with risk group classification in children with acute lymphoblastic leukemia. BMC cancer. 2013;13:452 10.1186/1471-2407-13-452 24088574 PMC3850706

[24] Wedemeyer C, Kauther MD, Hanenkamp S, Nuckel H, Bau M, Siffert W, et al BCL2-938C>A and CALCA-1786T>C polymorphisms in aseptic loosened total hip arthroplasty. European journal of medical research. 2009;14(6):250–5. 19541585 10.1186/2047-783X-14-6-250 PMC3352017

[25] Bachmann HS, Siffert W, Frey UH. Successful amplification of extremely GC-rich promoter regions using a novel 'slowdown PCR' technique. Pharmacogenetics. 2003;13(12):759–66. 14646694 10.1097/00008571-200312000-00006

[26] Rodriguez S, Gaunt TR, Day IN. Hardy-Weinberg equilibrium testing of biological ascertainment for Mendelian randomization studies. American journal of epidemiology. 2009;169(4):505–14. 10.1093/aje/kwn359 19126586 PMC2640163

[27] Adelani MA, Crook K, Barrack RL, Maloney WJ, Clohisy JC. What is the prognosis of revision total hip arthroplasty in patients 55 years and younger? Clinical orthopaedics and related research. 2014;472(5):1518–25. 10.1007/s11999-013-3377-9 24249534 PMC3971212

[28] Mahomed NN, Barrett JA, Katz JN, Phillips CB, Losina E, Lew RA, et al Rates and outcomes of primary and revision total hip replacement in the United States medicare population. The Journal of bone and joint surgery American volume. 2003;85-a(1):27–32. 12533568 10.2106/00004623-200301000-00005

[29] Yan Y, Hu J, Lu H, Wang W. Genetic susceptibility to total hip arthroplasty failure: a case-control study on the influence of MMP 1 gene polymorphism. Diagnostic pathology. 2014;9:177 10.1186/s13000-014-0177-9 25257555 PMC4180955

[30] Pan F, Hua S, Luo Y, Yin D, Ma Z. Genetic susceptibility of early aseptic loosening after total hip arthroplasty: the influence of TIMP-1 gene polymorphism on Chinese Han population. Journal of orthopaedic surgery and research. 2014;9(1):108. 25466591 10.1186/s13018-014-0108-1 PMC4324875

[31] Sundfeldt M, Carlsson LV, Johansson CB, Thomsen P, Gretzer C. Aseptic loosening, not only a question of wear: a review of different theories. Acta orthopaedica. 2006;77(2):177–97. 16752278 10.1080/17453670610045902

[32] Lucht U. The Danish Hip Arthroplasty Register. Acta orthopaedica Scandinavica. 2000;71(5):433–9. 11186396 10.1080/000164700317381081

[33] Karrholm J. The Swedish Hip Arthroplasty Register (www.shpr.se). Acta orthopaedica. 2010;81(1):3–4. 10.3109/17453671003635918 20170435 PMC2856196

[34] Gorenoi V, Schonermark MP, Hagen A. Arthroplasty register for Germany. GMS health technology assessment. 2009;5:Doc13 10.3205/hta000075 21289900 PMC3011280

[35] Makela KT, Matilainen M, Pulkkinen P, Fenstad AM, Havelin LI, Engesaeter L, et al Countrywise results of total hip replacement. An analysis of 438,733 hips based on the Nordic Arthroplasty Register Association database. Acta orthopaedica. 2014;85(2):107–16. 10.3109/17453674.2014.893498 24650019 PMC3967250

[36] Makela KT, Eskelinen A, Pulkkinen P, Paavolainen P, Remes V. Total hip arthroplasty for primary osteoarthritis in patients fifty-five years of age or older. An analysis of the Finnish arthroplasty registry. The Journal of bone and joint surgery American volume. 2008;90(10):2160–70. 10.2106/JBJS.G.00870 18829914

[37] MacInnes SJ, Fenstad A, Michael A, Buckle C, Furnes O, Hallan G, et al. Using a National Joint Register Dataset and Postal Methodology to Develop a Large DNA Archive for Musculoskeletal Disease. ORS 2014 Annual Meeting. 2014;PS2:Poster 1432.

